# Dopamine 2 Receptor Activation Entrains Circadian Clocks in Mouse Retinal Pigment Epithelium

**DOI:** 10.1038/s41598-017-05394-x

**Published:** 2017-07-11

**Authors:** Kenkichi Baba, Jason P. DeBruyne, Gianluca Tosini

**Affiliations:** Circadian Rhythms and Sleep Disorders Program, Neuroscience Institute, and Department of Pharmacology & Toxicology, Morehouse School of Medicine, Atlanta, GA 30310 USA

## Abstract

Many of the physiological, cellular, and molecular rhythms that are present within the eye are under the control of circadian clocks. Experimental evidence suggests that the retinal circadian clock, or its output signals (e.g., dopamine and melatonin), may contribute to eye disease and pathology. We recently developed a retinal pigment ephithelium (RPE)-choroid preparation to monitor the circadian clock using PERIOD2 (PER2)::LUC knock-in mouse. In this study we report that dopamine, but not melatonin, is responsible for entrainment of the PER2::LUC bioluminescence rhythm in mouse RPE-choroid. Dopamine induced phase-advances of the PER2::LUC bioluminescence rhythm during the subjective day and phase-delays in the late subjective night. We found that dopamine acts exclusively through Dopamine 2 Receptors to entrain the circadian rhythm in PER2::LUC bioluminescence. Finallly, we found that DA-induced expression of core circadian clock genes Period1 and Period2 accompanied both phase advances and phase delays of the RPE-choroid clock, thus suggesting that – as in other tissues – the rapid induction of these circadian clock genes drives the resetting process. Since the RPE cells persist for the entire lifespan of an organism, we believe that RPE-choroid preparation may represent a new and unique tool to study the effects of circadian disruption during aging.

## Introduction

The presence of a retinal circadian clock in mammals was first reported in the late 90s^1, 2^ and several studies have now demonstrated that many of the physiological, cellular and molecular rhythms within the retina are under the control of cell-autonomous intrinsic circadian clocks^3, 4^. Emerging experimental evidence also indicates the presence of circadian clocks in other ocular structures such as cornea and retinal pigment epithelium (RPE) that are involved in the control of many ocular circadian rhythms (e.g., intraocular pressure, photoreceptor disk shedding and phagocytosis, axial chamber length, choroidal volume, corneal curvature and cornea thickness)^3, 4^.

The current understanding of the circadian system within the mouse eye has been greatly advanced by the use of the PERIOD2::Luciferase (PER2::LUC) mouse that allows real-time monitoring of clock functions^5^. Using this mouse, several laboratories have been able to demonstrate that circadian clocks are present not only in the retina^6, 7^, but also the cornea^5, 8^ and that the (RPE)^9^. Hence the mouse eye is a bona fide circadian system containing circadian oscillators in several different cell types and cell types, all of which must be synchronized for the system to function correctly. Although we do not know which of these clocks function as the ‘master’ circadian oscillator within the eye, we know that only the retina responds directly to light^1, 2, 7, 9^, and the circadian rhythm in PER2::LUC bioluminescence in the retina is likely to be entrained via OPN 5^10^. Therefore, the retina must communicate photo-entrainment signals within the retina and to the rest of the ocular circadian system in order to maintain the clocks and synchrony in different non-photosensitive ocular structures. Among the several neurotransmitters/hormones present in the retina, melatonin (MLT) and dopamine (DA) have emerged as two likely candidates to transmit this signal throughout the eye. DA functions as a rhythmic humoral signal for light, producing light-adaptive physiology^7, 11^, and MLT is a rhythmic signal of darkness and has dark-adaptive effects^12, 13^. Consistent with this hypothesis, we have recently shown that the PER2::LUC bioluminescence rhythm in the mouse cornea can be entrained by melatonin via activation of Melatonin Receptor 2^8^.

The aim of the present study was to investigate whether MLT or/and DA play a role in the entrainment of the PER2::LUC bioluminescence rhythm in RPE. Our data indicate that DA - via Dopamine 2 Receptors (D_2_R) present in RPE - entrains circadian PER2::LUC bioluminescence rhythms in the mouse RPE.

## Results

### Dopamine, but not melatonin, phase-shifts the circadian rhythm in PER2::LUC bioluminescence

**Figure 1:**
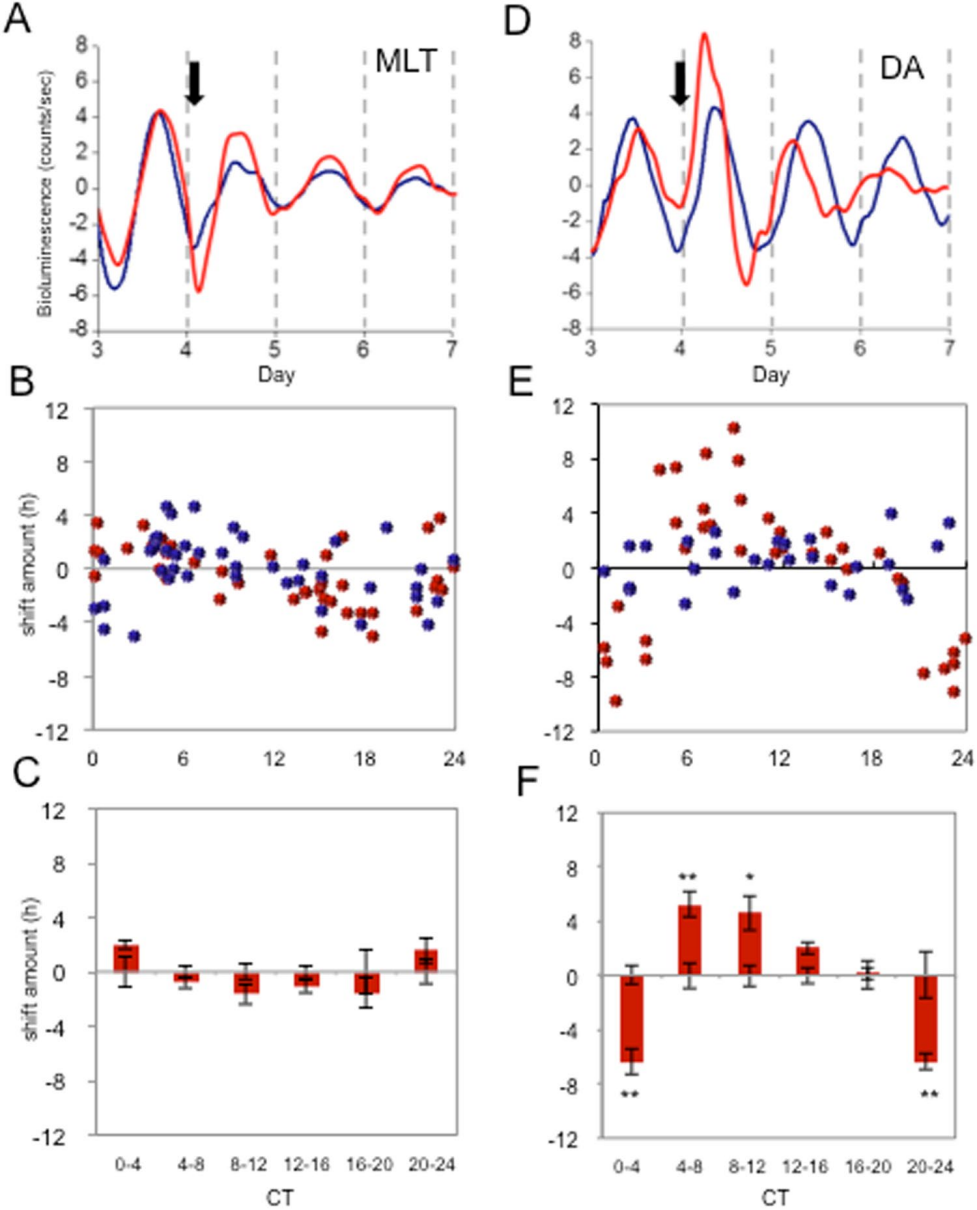
Dopamine phase-shifts PER2::LUC bioluminescence rhythms in mouse RPE-choroid. DA or MLT were added to the culturing media after the third peak of the RPE-choroid PER2::LUC bioluminescence rhythm. The representative data shows application of MLT at CT 14 did not phase-shift the RPE-choroid bioluminescence PER2::LUC rhythm (**A**). On the other hand, DA application at CT 8 phase-shifted the RPE-choroid bioluminescence rhythm (**D**). The blue traces indicate controls (vehicle treated) while red traces indicate MLT (**A**) or DA (**D**) treated RPE-choroid cultures. The black arrows indicate time of the drug or vehicle (Veh) treatments (**A** and **D**). The amount of phase-shift for each individual RPE-choroid rhythm was plotted to create a PRC (**B** and **E**). Blue circles indicate cultures treated with Veh and red circles indicate culture treated with either MLT (**B**) or DA (**E**). Data were divided into 6 bins at 4-hour intervals for statistical analysis. Data were then used to calculate the phase change of MLT or DA versus their vehicle controls. Bars show the mean amount of phase change from controls and error bars show ±SEM for experimental groups. Error bars from x axis show ±SEM for control groups. MLT did not phase-shift the RPE-choroid PER2::LUC bioluminescence rhythm (n = 4–14 for each bin, Two way ANOVA, p > 0.1, (**C**) whereas DA significantly phase-shifted PER2::LUC rhythm (Two way ANOVA following Tukey tests, *p < 0.05, **p < 0.01, n = 6–8 for each bin, (**F**).

To determine whether DA and/or MLT can entrain RPE rhythms, we applied either compound to PER2::LUC RPE-choroid cultures at various times and measured the resultant shift in rhythm phase. We found that, in contrast to the cornea clock^8^, the RPE clock was not reset by 100 nM MLT (Fig. 1A–C) when applied at any time of day. In contrast, 100 μM DA shifts the clock forward (advance) or backward (delay) by up to 8–12 hours, depending on the time it was administered (Fig. 1D–F). To aid in analysis, we binned individual phase-shifts into 4-hour phase intervals for statistical analysis. Two-way ANOVA revealed a significant interaction between treatment (vehicle or drug) and time of treatment in the DA treated cultures (p < 0.001; Fig. 1F), but not in the MLT treated cultures (p > 0.05; Fig. 1C). Overall, DA phase-delayed the RPE clocks by ~6.4 hours when given in an 8-hour window centered on a circadian “dawn” (CT0), phase advanced the RPE clock by approximately 5 hours when given throughout the rest of the circadian “day” (CT 4–12), and produced little effect on phase when given during the first 8 hours of the circadian “night” (CT 12–20) (Fig. 1F). Overall, these results suggest that DA, and not MLT, may be a circadian entraining signal for RPE in the retinal circadian system.

### Activation of D_2_R Signaling in the RPE Phase-shifts the Circadian Rhythm in PER2::LUC Bioluminescence

**Figure 2:**
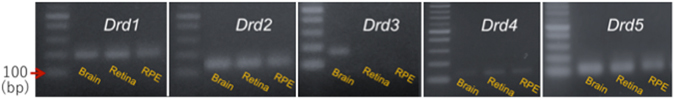
Expression of dopamine receptors in the brain, retina, and RPE. Agarose gel electrophoresis of PCR amplicons specific to D_1_R, D_2_R, D_3_R, D_4_R or D_5_R transcripts in brain, retina and RPE. D_3_R mRNA was present in the brain (positive control), but was not amplified in the retina (negative control) or RPE. The electrophoresis bands matched the expected amplicon size.

**Figure 3:**
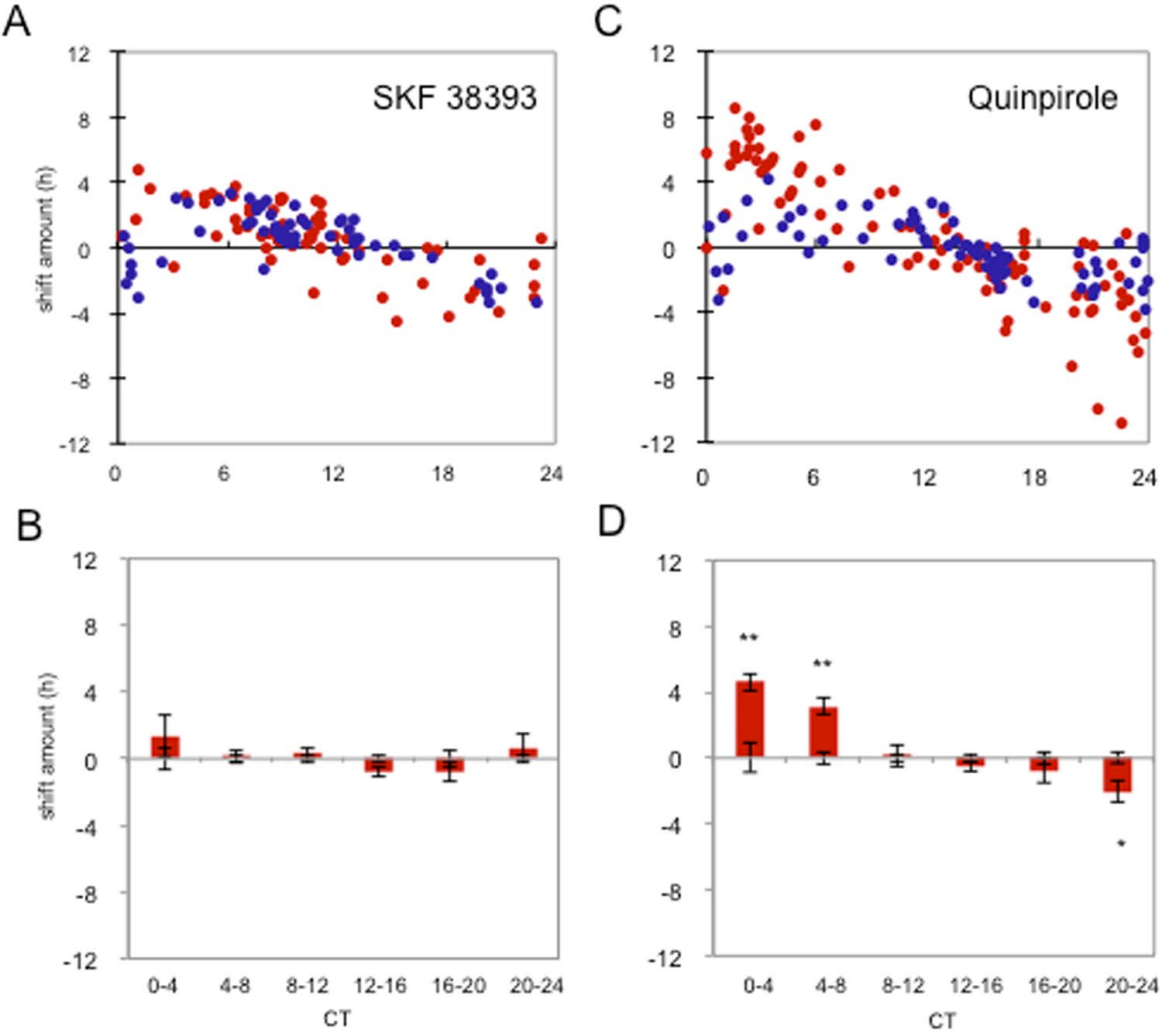
Effects of D_1_-like agonist (SKF38393) and D_2_-like agonist (Quinpirole) on PER2::LUC bioluminescence rhythm. 50 µM of SKF38393 did not phase-shift the PER2::LUC bioluminescence rhythm (**A** and **B**), whereas quinpirole significantly phase-advanced PER2::LUC bioluminescence rhythm at CT 0–4 and CT 4–8 hrs and phase-delayed when applied at CT 20–24 (**C** and **D**). Blue circles indicate cultures treated with vehicle and red circles indicate culture treated with active compounds. Data were divided to 6 bins at 4-hour intervals for statistical analysis (Two-way ANOVA following Tukey tests, *p < 0.05). n = 3–21 for each bin (**B** and **D**). Data were then used to calculate the phase change of drug treated versus their vehicle controls. Bars show the mean amount of phase change from controls and error bars show ±SEM for experimental groups. Error bars from x axis show ±SEM for control groups.

We then investigated which of the different DA receptors were responsible for phase-shift of the PER2::LUC circadian rhythm. There are five DA receptors in mammals, which are classified into D_1_-like (D_1_R, D_5_R) or D_2_-like (D_2_R, D_3_R, D_4_R) based on similar pharmacological profiles and coupling to second-messenger cascades^14, 15^. We found that all but the D_3_R s are detectably expressed in RPE (Fig. 2). We therefore investigated whether general D_1_-like or D_2_-like receptor agonists (SKF38393 or quinpirole, respectively) produced phase-response curves comparable to DA (Fig. 1F). We found that SKF38393 could not induce a phase-shift, regardless of when it was given (Fig. 3A and B). However, quinpirole administration phase-shifted the rhythm in a manner that was similar to that of DA (Fig. 3C). Quinpirole significantly phase-delayed PER2::LUC rhythms by 2.05 ± 0.65 hrs. when applied at CT 20–24 h (p < 0.05) and significantly phase-advanced PER2::LUC rhythms by 4.60 ± 0.54 hrs. and 3.16 ± 0.55 hrs when applied at CT 0–4 h and CT 4–8 h, respectively (p < 0.05; Fig. 3C,D). Overall, these data suggest that DA acting on D_2_Rs is sufficient to reset RPE rhythms.

**Figure 4:**
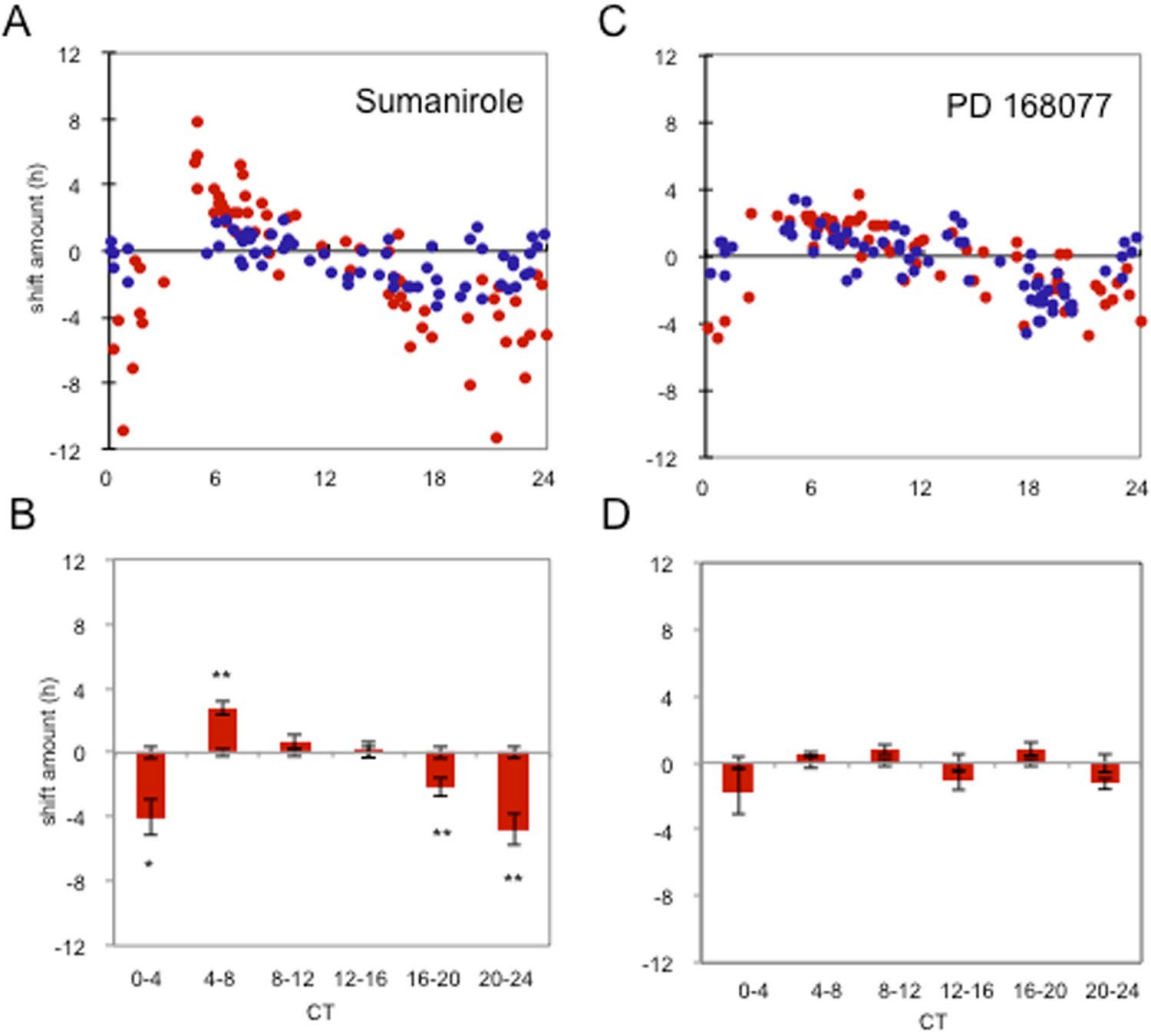
Effects of D_2_R (Sumanirole) and D_4_R (PD168077) agonists on the PER2::LUC bioluminescence rhythm. Sumanirole of 1 µM significantly phase-delayed PER2::LUC bioluminescence rhythm at CT 0–4, CT 16–20, and CT 20–24 h. Sumanirole significantly phase-advanced the bioluminescence rhythm at CT 4–8 (**A** and **B**). PD166077 did not phase-shift the PER2::LUC bioluminescence rhythms (**C** and **D**). Blue circles indicate cultures treated with vehicle and red circles indicate culture treated with active compounds (**A** and **C**). Data were divided to 6 bins at 4-hour intervals for statistical analysis (Two-way ANOVA following Tukey tests, *p < 0.05) n = 6–21 for each bin (**B** and **D**). Data were then used to calculate the phase change of drug treated versus their vehicle controls. Bars show the mean amount of phase change from controls and error bars show ±SEM for experimental groups. Error bars from x axis show ±SEM for control groups.

We next investigated which of the D_2_-like receptor subtypes where responsible for the observed phenomenon. Since D_3_R mRNA was undetectable in the RPE (Fig. 2), we focused on discriminating between D_2_R and D_4_R. Administration of Sumanirole, a D_2_R specific agonist, at various times of day shifted the clock in a manner similar to DA and quinpirole (Fig. 4A and B), whereas administering PD168077, D_4_R receptor specific agonist, did not phase-shift PER2::LUC rhythms in RPE-choroid (Fig. 4C and D). Thus, it appears that activation of D_2_Rs is sufficient to reset the RPE clock.

### Removal of D_2_R Signaling Prevents DA-induced Phase-shift of PER2::LUC Bioluminescence Rhythm in RPE

**Figure 5:**
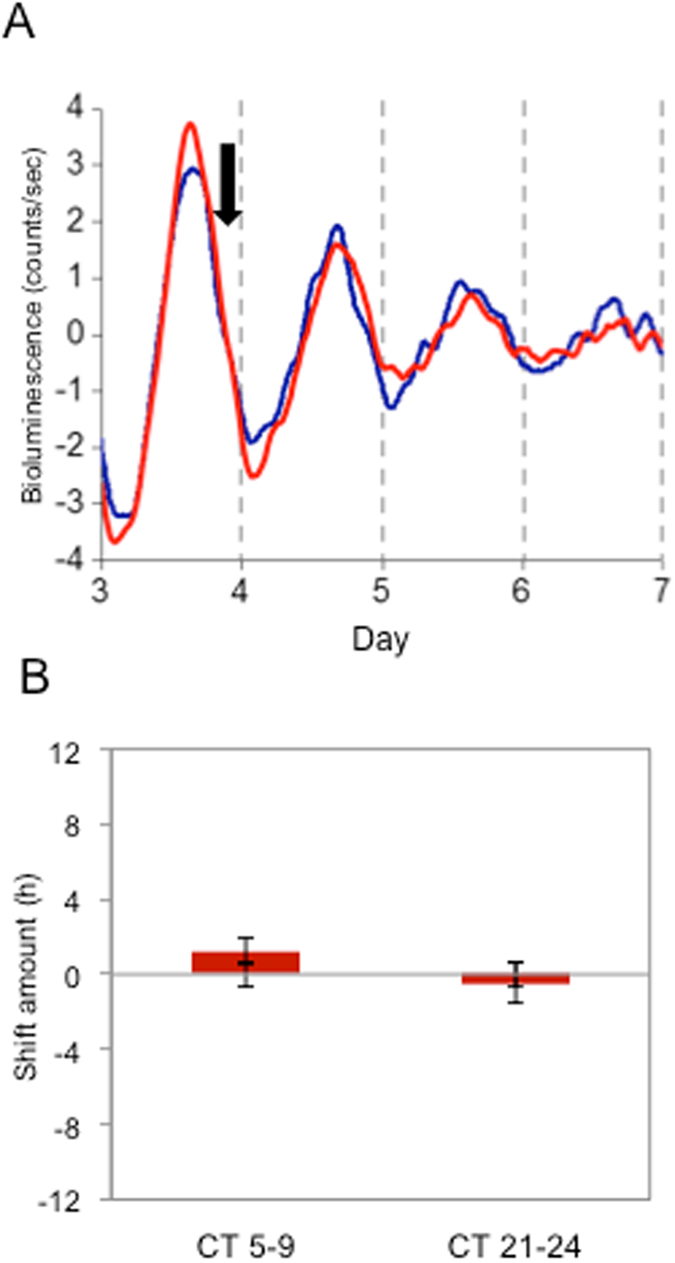
DA did not induce phase-shifts in the RPE-choroid of PER2::LUC bioluminescence rhythms in D_2_R^−/−^PER2::LUC mice. Representative example shows that DA treatment at CT 24 did not phase-shift D_2_R^−/−^PER2::LUC bioluminescence rhythm (**A**). The blue trace indicates a control (Veh treated) and red trace indicates a DA treated RPE-choroid culture. The black arrow indicates time of the drug or vehicle treatment (**A**). DA treated RPE-choroid cultures at CT 5–9 and at CT 21–24 were averaged for statistical analysis. Data were used to calculate the phase change of D_2_R^−/−^ versus controls. Bars show the mean amount of phase change from controls and error bars show ±SEM for experimental groups. Error bars from x axis show ±SEM for control groups. No phase-shifts were observed in the D_2_R^−/−^PER2::LUC RPE-choroid rhythms at either time points (**B**) *t*-test, n = 6 for DA and Veh for each time point).

We next tested if D_2_R was required for DA’s action on the RPE-clock by determining if DA could reset the clock of RPE-choroid from D_2_R-deficient PER2::LUC (*Drd2* ^−/−^; PER2::LUC) mice. The RPE-choroid tissues obtained from D_2_R^−/−^; PER2::LUC mice showed robust circadian rhythms (Fig. 5A) that were comparable in phase and periods to wild-type PER2::LUC controls (phases: 16.54 ± 0.20 hrs vs. 15.67 ± 0.53 hrs, periods: 23.88 ± 0.12 hrs vs. 23.68 ± 0.11 hrs p > 0.05, *t*-test, control vs. D_2_R^−/−^ respectively). We then treated RPE-choroid with 100 μM of DA at circadian times when DA induces phase advances or delays. As expected, D_2_R-deficiency eliminated DA-induced phase-shifts of RPE-choroid bioluminescence rhythms (Fig. 5A and B), confirming that DA is signaling RPE clocks exclusively via the D_2_R.

### DA Induces *Period1* and *Period2* Gene Expression in the RPE

**Figure 6:**
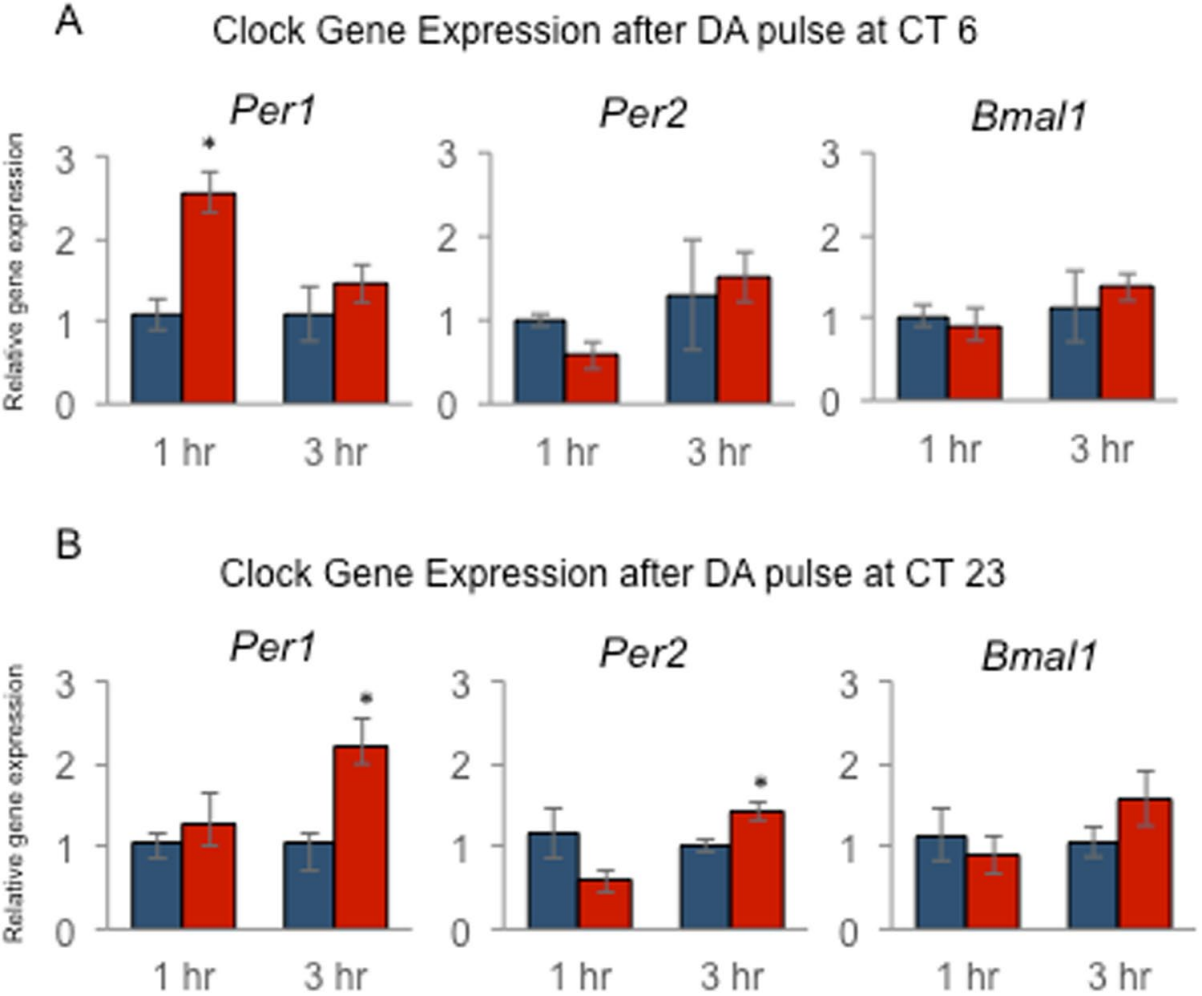
DA treatment increases *Per1* and *Per2* mRNA in RPE-choroid. RPE-choroid cultures were prepared and treated with DA or vehicle at ZT6 (**A**, advance) or ZT 23 (**B**, delay) as indicated above, followed by collection for Q-PCR analysis of the indicated mRNAs at 1 or 3 hour intervals. Expression data were normalized using 18S, and are plotted relative to vehicle controls. Blue bars indicate mean ± SEM of vehicle control. Red bars indicate mean ± SEM of DA treated. *Indicates p < 0.05 (*t-*test) compared to vehicle controls (n = 3–6 in each group).

Previous studies have shown that acute induction of *Period1* (*Per1*) and *Period2* (*Per2*) gene expression mediates phase-shifting of the circadian clock^16–18^. Hence, we decided to measure acute induction of *Per1* and *Per2* expression in cultured RPE-choroids after either one hour or three hours of DA treatment. DA applied at CT 6 to cause a phase advance (Fig. 1) significantly induced *Per1* expression (Fig. 6A, *t*-test, p < 0.05). Interestingly, *Per1* expression was only elevated at 1 hour after the DA treatment, returning to baseline 3 hours after the DA treatment (Fig. 6B). DA applied at CT 6 did not significantly alter *Per2* or *Bmal1* mRNA levels (Fig. 6B, *t*-test, p > 0.05 for both cases). DA administered at CT23, when it induces phase-delays (Fig. 1), did not significantly change levels of *Per1*, *Per2* and *Bmal1* mRNAs at 1-hr (Fig. 6C, *t*-test, p > 0.05), but significantly induced *Per1* and *Per2* mRNA 3-hrs after the DA pulse (Fig. 6D; *t*-test, p < 0.05). No changes were observed in *Bmal1* mRNA levels (Fig. 6C,D; *t*-test, p > 0.05). Thus, taken together, our results suggest that D_2_R activation mediates clock reset by acutely inducing expression of *Per1* and *Per2*.

## Discussion

Accumulating evidence indicates that disruption of circadian rhythms due to genetic mutations or environmental factors contributes to the development of many diseases and premature aging. Indeed circadian disruption has been associated with numerous immune, inflammatory, and metabolic disorders^19–21^. Experimental evidence also suggests that the retinal circadian clock, or its output signals (e.g., DA and MLT), may contribute to eye disease and pathology. For example, diabetes is associated with reduced clock gene expression in the retina^22^, and circadian disruption recapitulates diabetic retinopathy in mice^23^. Removal of *Bmal1* from the neural retina alters inner retinal function^24^ and a recent paper reported that mice lacking *Per1* and *Per2* show significant alteration in the distribution of cone photoreceptors^25^. Finally a series of studies have implicated the clock genes *Rev-erb*α and *Rora* in retinal functioning^26, 27^ and age-related macular degeneration^28^. Similarly, disruption of DA or MLT signaling in the mouse retina greatly affects retinal physiology and retinal cell viability^11–13, 29–32^.

Disruption of the daily rhythm of RPE phagocytosis impairs retinal and/or RPE functions. RPE of mice lacking αvβ5 integrin (β5^−/−^ mice) fail to show a circadian burst of phagocytic activity one of two hours after light onset. Also, during the aging process, β5^−/−^ mice lose both cone and rod photoreceptors faster than control mice^33^. The mechanism controlling the daily rhythm in RPE phagocytosis appears to be located in the RPE^34^ and is likely to be directly controlled by the circadian clock located in this tissue^9^.

Previous studies have also reported that DA receptors are involved in the regulation of rhythmic RPE functions. For example, inhibition of DA synthesis during the early part of the light phase induced a significant reduction of disk shedding and phagocytosis^35^. In addition, mice whose dopaminergic neurons have been destroyed by 1-methyl-4-phenyl-1,2,3,6-tetrahydropyridine (MPTP) accumulate a large number of residual bodies in the RPE^36^. It has also been reported that dopamine and a D_1_-like activation decrease phagocytosis by RPE cells^37^. However, the presence of dopamine receptors in the mammalian RPE is still controversial^38^.

Our data indicated that DA can phase-shift in a time dependent manner the circadian rhythm in PER2::LUC bioluminescence (Fig. 1), and that D_1_R, D_2_R, D_4_R and D_5_R mRNAs are present in the mouse RPE (Fig. 2), albeit only in pharmacological activation of D_2_R induced a phase-shift in the PER2::LUC bioluminescence rhythm (Figs 3 and 4). The timing of quinpirole induced phase shifts was advanced compared to DA or Sumanirole. However, this is probably due to a difference in affinity of these ligands for D_2_R^39–41^ and not due to involvement of other DA receptors as removing the D_2_R receptor completely abolished any resetting responses of the RPE clock in response to DA (Fig. 5).

A number of studies support a model in which the rapid induction of the circadian clock genes *Per1* and *Per2* drives the resetting process^42, 43^. The induction of *Per1* mRNA in the suprachiasmatic nucleus of the hypothalamus following a photic stimulus is thought to be driven by activation of cAMP response element-binding protein (CREB) located in the promoter region of the *Per1* gene^44, 45^. D_2_-like receptors are negatively coupled to adenylyl cyclase and thereby lead to a decrease of cAMP levels. Thus, it is unlikely that D_2_R activation induces *Per1* mRNA via the cAMP signaling pathway. Surprisingly our data (Fig. 6) indicates that *Per1* mRNA levels were significantly increased 1-hr after DA treatment at CT 6 when DA phase-advanced the PER2::LUC rhythm. In comparison, *Per1* and *Per2* expression was induced 3 hrs after the treatment at CT 23 when DA phase-delayed the PER2::LUC rhythm. These data agree well with previous studies in the mouse in which it was reported that mice lacking *Per1* did not show any phase-advance after a pulse of light, whereas mice lacking *Per2* did not show any delays after a pulse of light^43^. Our results are also consistent with the findings of another investigation reporting that a light pulse during the delay zone of the PRC induces the expression of *Per1* and *Per2* genes, whereas a light pulse during the advance zone of PRC increases only *Per1* ^46^. Thus experimental evidence agrees well with our results and supports the hypothesis that DA – via D_2_R activation – can induce *Per1* and *Per2* expression. The molecular mechanism by which DA via D_2_R activation induces *Per1* and *Per2* is unknown and further studies will be required to address this important issue.

Finally, it is worth mentioning that the RPE is composed of a single cell type and persists for the entire lifespan of an organism. Thus, a RPE-choroid preparation may represent a new and unique tool to study the impact of circadian clock function and disruption on cellular biology and longevity over a full lifespan.

## Methods

### Animals

This study used PER2::LUC mice (C57Bl/6) of 3 to 6 months in age. PER2::LUC mice were crossed with Dopamine 2 Receptor knock- out (D_2_R^−/−^) to produce D_2_R^−/−^ PER2::LUC mice. The original D_2_R^−/−^ were purchased from Jackson Laboratory (strain *Drd2* ^*tm1Low*^/*J*). All mice used in this study were raised at Morehouse School of Medicine in 12 h light and 12 h dark with lights-on (Zeitgeber Time; ZT 0) at 06:00 and lights off (ZT 12) at 18:00 h local time. Water and food were available ad libitum. Light was supplied with fluorescent tubes and the light intensity ranged from 200 to 400 *lux* at cage level. Animal experimentation was carried out in accordance with the National Institutes of Health Guide on the Care and Use of Laboratory Animals and the ARVO Statement for the Use of Animals in Ophthalmic Vision Research, and was approved by the Institutional Animal Care and Use Committees of Morehouse School of Medicine.

### Tissue Culture Preparation and Measurement of Bioluminescence

Mice were anesthetized with CO_2_ and then sacrificed by cervical dislocation. The eyes were removed and the retina and RPE-choroid cup were carefully separated under a dissecting microscope. The eye cup containing the RPE-choroid was flattened by four radical incisions, and then placed on the culture membrane (Millicell-CM, PICM030-50, Millipore, Billerica, MA) in a 35 mm Petri dish with 1.2 ml of 199 medium (Cambrex, Walkersville, MD) containing 0.1 mM D-Luciferin K salt (Molecular Imaging Products, Bend, OR). Dishes were sealed and kept at 37 °C. The cultures were prepared under fluorescent tubes between Zeitgeber Time (ZT, lights on at ZT0) 8–10. The bioluminescence emitted from the RPE was measured with photomultiplier tubes (Lumicycle®; Actimetrics, Wilmette, IL).

### Quantitative Real Time RT-PCR (Q-PCR)

RPE-choroid cup culture dishes were prepared as described above and kept in the incubator at 37 °C. After 3 days of culture, dishes were taken from the incubator and either dopamine (final concentration of 100 µM) or vehicle was added to the culture medium at either CT 6 or CT23 and culture dishes were returned to the incubator. At 1 hour or 3 hours after the dopamine treatment, culture tissues were collected from dish and subjected for RNA extraction using Trizol (Thermo fisher scientific, MA). Q-PCR was performed using the CFX96 Touch Real-Time PCR Detection System (Bio-Rad Laboratories, Hercules, CA, USA) using iQ SYBR Green Supermix (Bio-Rad Laboratories). All data for individual genes were normalized to 18S, and are plotted relative to average levels in vehicle treated control samples collected in parallel (n = 6 cultures for DA groups, n = 3 cultures for vehicle controls).

The details of procedures and primer sequences of *Per1*, *Per2*, *Bmal1 and 18S* are reported in Hiragaki *et al*.^47^. The primers used to amplify dopamine receptors expression are as follows: *Drd1* (193 bp) forward 5′-cagccttcatcctgattagcgtag-3′ reverse 5′-cttatgagggaggatgaaatggcg-3′, *Drd2* (148 bp) forward 5′-tgccttcgtggtctactcct-3′ reverse 5′-tgccttcgtggtctactcct-3′, *Drd3* (116 bp) forward 5′-ccagacacatggagagctga-3′ reverse 5′-aggagttccgagtcctctcc-3′, *Drd4* (131 bp) forward 5′-cgtctctgtgacacgctcat-3′ reverse 5′-cactgaccctgctggttgta-3′, and *Drd5* (109 bp) forward 5′-catccatcaagaaggagaccaagg-3′ reverse 5′-cagaagggaaccatacagttcagg-3′.

### Drug treatments

Dopamine (Sigma) was dissolved (100 µM final concentration) in PBS and ascorbic acid (50 µM final concentration) was added to prevent oxidation. Melatonin (Sigma) was first dissolved in ethanol (8 mg/ml) and then was diluted with PBS (100 nM final concentration). SKF 38393 (Sigma) was dissolved in PBS (50 µM final concentration), and Quinpirole (Tocris) was dissolved in PBS (50 µM final concentration). Sumanirole (Tocris) was first dissolved in distilled water (32 mg/ml) and then diluted to 1 mM (1 µM final concentration) with PBS. PD168077 was first dissolved in DMSO (45 mg/ml) then diluted with PBS to 1 mM (1 µM final concentration). These concentrations were selected on the basis on the compound affinity for each of receptors. After 3 to 4 days of bioluminescence recording, the culture dishes containing a RPE-choroid cup were gently removed from the Lumicycle® and either 1.2 μl (DA, Sumanirole and PD168077) or 6 μl (SKF and Quinpirole) volumes of drug solutions or vehicles were added to the culture dishes. They were then re-sealed, returned to the Lumicycle® and placed in the same positions that they were occupying before the treatment. The culture dishes were kept in the Lumicycle® until the end of the experiment without a drug washout.

### Analysis of Phase-shifts

Bioluminescence recordings emitted from RPE-choroid cultures were detrended by a 24-hour moving average subtraction method and smoothed by a 2-hour moving average. The circadian time (CT) of bioluminescence recordings were determined by the projection of the light cycle to which the mice were exposed (CT 12 = lights off). The circadian peak phase was determined as the highest point of the curve picked by Origin® (Origin Lab, Northampton, MA) software. The amount of phase-shift (in hours.) was calculated by comparing the regression lines fitted to the circadian peaks before and after treatment. Phase-shifts in an individual culture dish were plotted as the phase response curve. Data were then averaged in 4 hr bins: CT 0–4, CT 4–8, CT 8–12, CT 12–16, CT 16–20, CT 20–24 and normalized with respective vehicle control groups^6^. Two-way ANOVA with a *post hoc* Tukey test was performed to compare the difference between experimental groups and time points.
